# Dedicated preparation for in situ transmission electron microscope tensile testing of exfoliated graphene

**DOI:** 10.1007/s42649-019-0005-5

**Published:** 2019-04-29

**Authors:** Kangsik Kim, Jong Chan Yoon, Jaemin Kim, Jung Hwa Kim, Suk Woo Lee, Aram Yoon, Zonghoon Lee

**Affiliations:** 1 School Materials Science and Engineering, Ulsan National Institute of Science and Technology (UNIST), Ulsan Metropolitan City, 44919 Republic of Korea; 2 Center for Multidimensional Carbon Materials, Institute for Basic Science (IBS), Ulsan Metropolitan City, 44919 Republic of Korea

**Keywords:** Exfoliated graphene, In situ TEM, Uniaxial tensile testing, Dry transfer, Crack propagation

## Abstract

Graphene, which is one of the most promising materials for its state-of-the-art applications, has received extensive attention because of its superior mechanical properties. However, there is little experimental evidence related to the mechanical properties of graphene at the atomic level because of the challenges associated with transferring atomically-thin two-dimensional (2D) materials onto microelectromechanical systems (MEMS) devices. In this study, we show successful dry transfer with a gel material of a stable, clean, and free-standing exfoliated graphene film onto a push-to-pull (PTP) device, which is a MEMS device used for uniaxial tensile testing in in situ transmission electron microscopy (TEM). Through the results of optical microscopy, Raman spectroscopy, and TEM, we demonstrate high quality exfoliated graphene on the PTP device. Finally, the stress–strain results corresponding to propagating cracks in folded graphene were simultaneously obtained during the tensile tests in TEM. The zigzag and armchair edges of graphene confirmed that the fracture occurred in association with the hexagonal lattice structure of graphene while the tensile testing. In the wake of the results, we envision the dedicated preparation and in situ TEM tensile experiments advance the understanding of the relationship between the mechanical properties and structural characteristics of 2D materials.

**Electronic supplementary material:**

The online version of this article (10.1007/s42649-019-0005-5) contains supplementary material, which is available to authorized users.

## Introduction

Since graphene was first prepared from graphite via mechanical exfoliation, so called Scotch tape method in 2004, graphene with a distinctive hexagonal lattice structure has attracted intensive attention because of its extraordinary properties (Novoselov et al. 2004; Geim and Novoselov 2007). Among them, the electrical properties of graphene are already known to exhibit unusual characteristics such as the formation of a Dirac point and high carrier mobility with massless particles (Zhang et al. 2005; Novoselov et al. 2005). These properties indicate that graphene can feasibly be used in miniaturized electronic devices (Bala and Marwaha 2016; Kyeremateng et al. 2017; El-Kady and Kaner 2013). The mechanical properties of graphene are also widely known to exhibit unusual characteristics including extremely high in-plane stiffness. Therefore, graphene might be used in various industrial fields (Chen et al. 2008; Zhao et al. 2010; Rafiee et al. 2009). For these reasons, graphene has attracted much attention in the field of strain engineering. For example, the engineering of the internal strain in graphene led to superconductivity, quantizing pseudo-magnetic field, and zero-field quantum Hall effect (Guinea et al. 2010; Si et al. 2013; Levy et al. 2010). Expanding the applications of graphene in strain engineering, where these outstanding properties can be exploited, requires a detailed understanding of the relationship between the structural and mechanical properties of graphene.

Although extensive simulation studies of the relationship between the in-plane mechanical properties and structure of graphene have been conducted to better understand its superior mechanical properties, atomic-level experimental evidence has been lacking (Zhang et al. 2011; Min and Aluru 2011; Scarpa et al. 2009). The mechanical properties of graphene have not been explained in detail, and previous studies have focused only on stress and Poisson ratios against strain in the armchair or zigzag direction in graphene (Liu et al. 2007; Pei et al. 2010). No optimized values related to the structure of graphene have been deduced. Thus, studies that experimentally link the structural properties with the engineering applications of two-dimensional (2D) materials are needed.

In general, the mechanical properties of any materials can be practically measured by methods such as tension, compression, and bending tests. In particular, because of its 2D geometric characteristics, the most important mechanical test for graphene is uniaxial tensile testing. These tests directly correspond to the reaction of forces being applied to in-plane *sp*^2^ carbon–carbon bonds in graphene. Uniaxial tensile testing reveals the role of three fundamental factors—the Young’s modulus *E*, the Poisson’s ratio *ν*, and the intrinsic strength *σ*_int_—in determining the phenomenological mechanical properties of 2D materials. However, the uniaxial tension testing of atomically thin 2D materials by using conventional equipment is difficult. Numerous researchers have attempted to overcome these difficulties through various approaches. The mechanical properties of exfoliated graphene measured by atomic force microscopy (AFM) have been speculated to represent the mechanical properties of graphene (Lee et al. 2008; Lee et al. 2013). The Young’s modulus of monolayer graphene is approximately 1 TPa, the maximum stress is 130 GPa, and the defects in graphene are known to reduce the strength by approximately 50% (Mortazavi and Cuniberti 2014). However, the issue of whether this reduced strength corresponds to an indirect transformed value of indentation by the AFM tip and does not reflect empirical values is unsettled (Han et al. 2015). Furthermore, this approach does not address the structural viewpoint. Therefore, studies to directly measure the mechanical properties of 2D materials with their structural characteristics are needed. We proposed conducting mechanical tests through microelectromechanical systems (MEMS) devices to address these problems (Liao et al. 2017; Cao et al. 2016).

MEMS devices, in conjunction with transmission electron microscopes, have recently been used to simultaneously characterize both the mechanical properties and structures of 2D materials. However, transferring atomically thin 2D materials onto the selective area in a MEMS device for TEM analysis is difficult. Nanomaterials are typically transferred onto MEMS devices via a focused-ion beam (FIB) or by wet or dry transfer in the case of a graphene prepared by exfoliation or chemical vapor deposition (Gammer et al. 2016; Cao et al. 2015; Wang et al. 2015; Zhang et al. 2014). These processes are difficult to apply to 2D materials because of complicated problems such as unintended damage induced by the FIB and impurity problems associated with polymer dissolution. Furthermore, for the direct characterization of ideal strength, mechanical testing must be carried out on a graphene sheet exfoliated from highly ordered pyrolytic graphite, or Kish graphite, which is defect-free up to 1 mm. These problems strongly affect the reliability of expensive and sensitive MEMS devices. A process that is safer and less sensitive to MEMS devices and specimens is strongly desired.

In this work, we successfully transferred stable, clean, and free-standing exfoliated graphene to a push-to-pull (PTP) device, which is one of the MEMS devices used in uniaxial tensile testing, by using dry transfer with a gel material; we then conducted tensile testing on the exfoliated graphene. Optical microscopy and cross-sectional high-resolution transmission electron microscopy (HRTEM) images confirmed that our technique led to the successful transfer to the PTP device. HRTEM images and Raman spectra confirmed that the gel material did not influence the mechanical properties of the graphene. Furthermore, we studied the mechanical properties of exfoliated graphene via stress–strain (S–S) curves obtained by in situ TEM tensile testing. Crack propagation in graphene was also observed. Our developed technique enables the in situ TEM tensile testing of graphene at an atomic level.

## Methods/experimental

### Exfoliation and transfer process

We used natural graphite crystals (Kish graphite grade 200, Graphene Market). The graphite was sonicated with isopropyl alcohol (IPA) to thin the graphite and weaken the interactions between the thin flakes. The graphite was then heated in a box furnace (FB 1310 M, ThermoFisher) for 1 h at 500 °C to evaporate the IPA (Mag-isa et al. 2015). We obtained the few-layer and sub-millimeter flakes of graphite by repetitive pealing by using adhesive tape as usual and then transferred the flakes from the adhesive tape to a thin layer of gel material suspended on a polyethylene terephthalate (PET) film (PF film, Gel-Pak). Given that the adhesion of the PF film was far weaker than that of the Scotch tape, only few-layer graphene could be transferred onto the PF film. For few-layer exfoliated graphene transferred by PF film, it is important to distinguish the thickness that can be adequately observed by TEM. The optical contrast difference of exfoliated graphene with the PF film was determined using an optical microscope (LEICA, DM 4000 M), and the number of observed layers matched the layer number determined by AFM (Veeco, MultiMode V). We also used a Raman spectrometer (Witec, Alpha 300R) equipped with the 532 nm laser to confirm the quality of the exfoliated graphene on the PTP device. Pt deposition was conducted using a FIB (FEI, Quanta 3D FIB) to ensure that the exfoliated graphene was clearly adhered to the PTP device. To avoid Ga-ion-induced beam damage while using the FIB apparatus, we conducted the deposition by using only an electron beam generated at 10 kV and 16 nA. The exfoliated graphene was not exposed to the scanning electron microscope window to avoid electron beams during deposition.

### TEM analysis and data processing

The in situ TEM tensile testing of exfoliated graphene on a PTP device was performed in a Titan Double Cs corrected TEM (FEI, Titan cubed G2 60–300) at an acceleration voltage of 80 kV to reduce knock-on damage in the exfoliated graphene. The holder system used in the in situ experiments was a Hysitron PI 95 TEM PicoIndenter, and the flat punch probe pushed the semi-circular part of the PTP device to perform tensile testing. Given that the PTP device itself has an identical spring with high stiffness in the lateral direction, we calibrated the S–S curve against broken exfoliated graphene. ImageJ software was used to confirm the distribution of the gel material on the exfoliated graphene. We removed reflections related to the periodic graphene lattice in the fast Fourier transform (FFT) images of the HRTEM images, and the background was inversed in the FFT image to enhance the contrast of the gel material. The density of the gel material was measured manually.

## Results and discussion

**Fig. 1:**
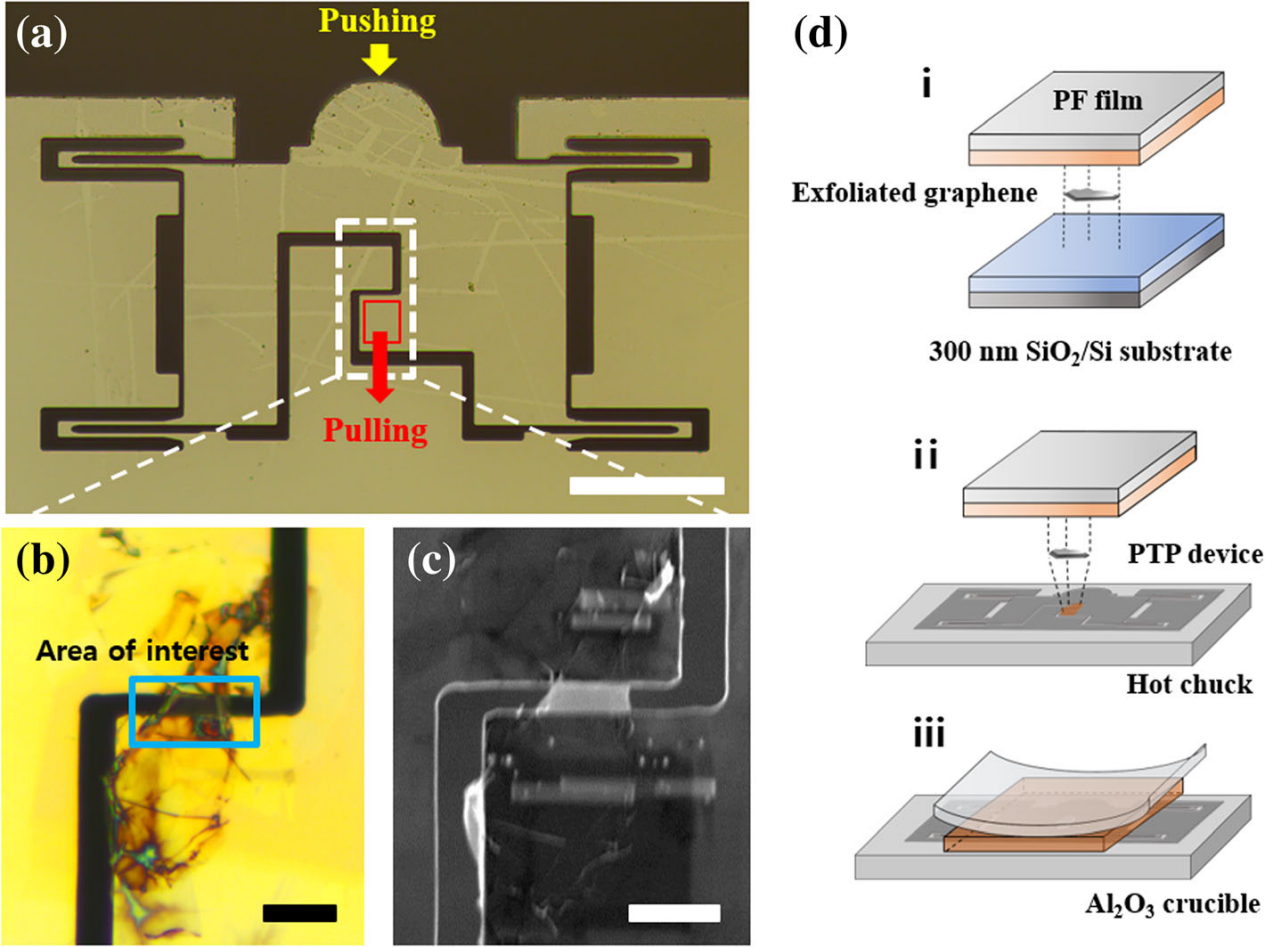
Transfer process of exfoliated graphene. **a** Optical image of the PTP device. The scale bar is 100 μm. **b** and **c** Optical microscope and SEM images of the exfoliated graphene on the PTP device after annealing and deposition of Pt grips, respectively. The scale bar is 5 μm. **d** Schematic showing the transfer exfoliated graphene to the PTP device

The PTP device capable of performing the uniaxial tensile testing of exfoliated graphene is shown in Fig. 1a. When the in situ TEM tensile testing was performed, a flat probe with a size of 100 μm pushed the hemispherical head in the PTP device. The lower area of the dashed box in Fig. 1a was pulled into the PTP device (Oh et al. 2014). The PTP device operates as its name, and the load value is calculated on the basis of a converted value obtained from electrostatic comb drive actuators in the flat probe. Specimens for uniaxial tensile testing on the PTP device were transferred to the dashed box shown in Fig. 1a, which includes an area of interest with a 2 μm gap. A schematic of the process used to transfer the exfoliated graphene onto the PTP device is shown in Fig. 1d. When we attempted to directly transfer to the PTP device via the stamping method by using a polydimethylsiloxane, we found that the exfoliated graphene is rarely transferred from the film to the interest area of the PTP device because the film was strongly adhered to the bonding material. Also, we found that the PTP devices are easily fractured after several dry transfer attempts through stamping, because the PTP devices are composed of brittle Si. Thus, we were compelled to develop a new method to transfer the exfoliated graphene to the PTP device.

We developed the following method for transferring specimens to sensitive PTP devices. First, before the transfer process, the exfoliated graphene peeled off by a PF film consisting of PET film and gel material was adhered to 300 nm SiO_2_ on a Si substrate for the measurement of the thickness of the exfoliated graphene via optical contrast difference between the substrate and exfoliated graphene with different layer numbers (Li et al. 2013). Considering that the engineering stress was calculated according to the thickness of the exfoliated graphene, it affected a sensitive value for determining the film’s mechanical properties. The difference in the optical contrast between the exfoliated graphene and supporting substrate, based on the results of AFM, was used to determine the thickness of the exfoliated graphene (Additional file 1: Figure S1). The exfoliated graphene on the PF film was then placed on our homemade position aligner with a hot chuck and manually controlled micromanipulators, which were used to align the specimen into the correct position (Additional file 2: Figure S2). After the sample was placed in the dashed box shown in Fig. 1a, it was heated in the hot chuck at 180 °C for approximately 1 h. The gel material under the PF film liquefied and permeated into the gap between the PTP device and exfoliated graphene (Pizzocchero et al. 2016). When we placed the sample on an Al_2_O_3_ crucible that was subsequently heated to 500 °C to remove the PET film for a few seconds, the PET film not melted but bent in response to the heat. The PET film was blown using air blow as it bent, leaving only the exfoliated graphene and the gel material on the PTP device. The remaining gel material was annealed in a box furnace at 500 °C for 10 min. Only the exfoliated graphene remained on the PTP device after the annealing process.

In our case, the *sp*^2^ bonding of the exfoliated graphene is stable up to 500 °C without forming additional defects (Nan et al. 2013). The in situ heating Raman spectra analysis confirmed that the gel material was evaporated when the PTP device was heated to 300 °C. Furthermore, the intensity ratio between the D and G peaks was approximately 0.09, thus indicating a low concentration of defects in exfoliated graphene (Additional file 3: Figure S3 and Additional file 4: Movie S1) (Venezuela et al. 2011; Lucchese et al. 2010; Cancado et al. 2011). In conclusion, we proved that defects were not introduced during the whole transfer process. The exfoliated graphene on the PTP device is shown in Fig. 1b. There is a color difference at the sides of the exfoliated graphene compared to the free-standing area because of the folded edge during this process. The electron beam in the FIB apparatus was subsequently used to deposit two Pt grips to fix the exfoliated graphene onto the PTP device for tensile testing as shown in Fig. 1c. We considered the two Pt grips as the initial length of gauge section when calculating the strain (Chen et al. 2015).

**Fig. 2:**
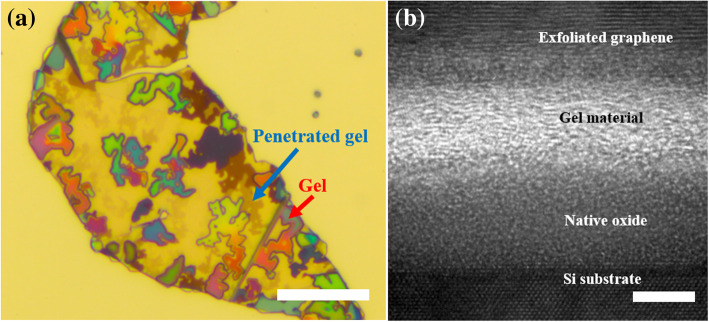
Results of liquefied gel material. **a** Optical image of liquefied gel material. The remaining gel is on the exfoliated graphene, whereas the rest of the gel penetrated under the exfoliated graphene. The scale bar is 20 μm. **b** Cross-sectional HRTEM image of gel material that penetrated under the exfoliated graphene. The scale bar is 5 nm

To further study how the exfoliated graphene was transferred from the PF film to the PTP device, the above-mentioned method was conducted on a Si substrate, which is the same material as the PTP device. Figure 2a is an optical image of the exfoliated graphene on the Si substrate, this figure shows that the PET film was removed, and the gel remained on or under the exfoliated graphene. In this image, the gel with different colors remains on the exfoliated graphene, whereas the gel with translucent colors is penetrated under the exfoliated graphene due to the melting of gel. To identify the gel material that penetrated under the exfoliated graphene, cross-sectional TEM analysis of the exfoliated graphene was conducted. As predicted, the gel was observed under the exfoliated graphene in the HRTEM image in Fig. 2b, which was confirmed by the energy-dispersive spectroscopy results (Additional file 5: Figure S4). No significant deterioration in the quality of exfoliated graphene was observed even after the gel was removed via an annealing process at 500 °C for 10 min (Additional file 6: Figure S5).

**Fig. 3:**
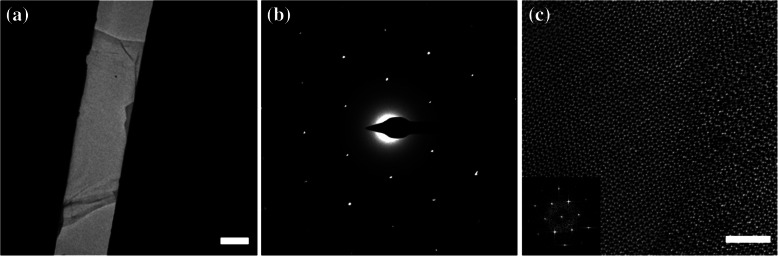
Quality check for the transferred graphene. **a** TEM image of the exfoliated graphene on a PTP device. The scale bar is 2 μm. **b** SADP image of the exfoliated graphene. **c** HRTEM image of exfoliated graphene. The right side in (**c**) is the remaining gel material. The inset of (**c**) is the FFT of the exfoliated graphene, which is the matched SADP of the exfoliated graphene. The scale bar is 2 nm

Figure 3a shows a TEM image of the exfoliated graphene onto a PTP device using our proposed method. We confirmed five layers of exfoliated graphene based on differences in optical contrast (Additional file 1: Figure S1). Unfortunately, even for the device prepared with our method, folded edges were observed in the TEM image. These folded edges could be induced by exfoliation or by an annealing process (Kim et al. 2011; Chen et al. 2014). In addition, as annealing process, the process of rolling or folding the exfoliated graphene is also considered because removal of the gel materials applied strain to graphene sheets. The hexagonal structure of the transferred graphene was confirmed through the HRTEM image and SADP in Fig. 3b and c. As shown in Figs. 1b and 3c, the gel material remained partially on the exfoliated graphene. To confirm the amount of remained gel materials on the exfoliated graphene, image processing was performed by removing the periodic components of the graphene reflections from the FFT image in the inset of Fig. 3c and Additional file 7: Figure S6 (Meyer et al. 2011). Additional annealing conducted even after the established transfer method to remove the remaining gel-material, was not effective as we expected, and left about 65% of the atomically-sharp, and clean graphene surface. Soft polymers, such as the gel material, are not expected to adversely affect the mechanical properties of strong and brittle exfoliated graphene (Zhang et al. 2014). Therefore, we proceeded the uniaxial tensile testing of the exfoliated graphene in PTP devices despite the remaining residue.

**Fig. 4:**
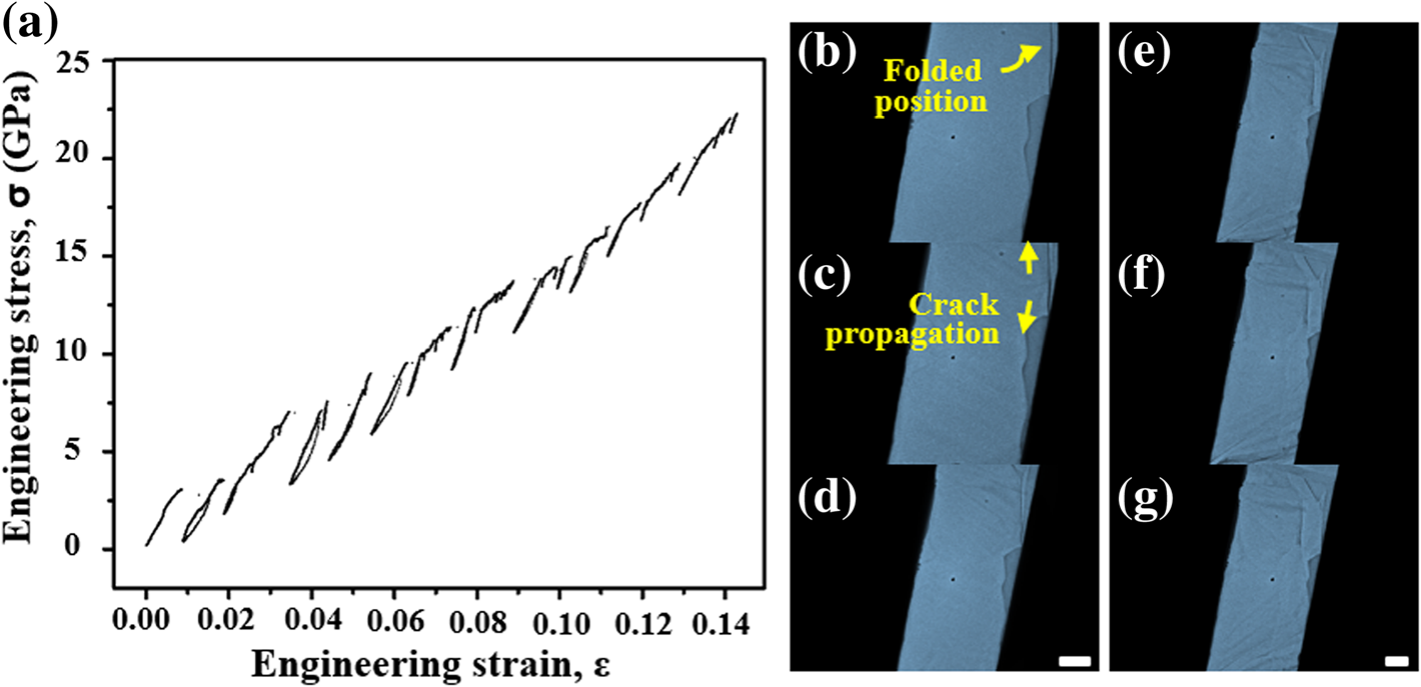
Result of in situ TEM tensile testing. **a** The stress–strain curve obtained via in situ TEM tensile testing of five-layer exfoliated graphene in a PTP device. **b-g** Image series of exfoliated graphene with crack propagation. The scale bar is 1 μm

Figure 4 and Additional file 8: Movie S2 show that the result of in situ TEM tensile tests of the exfoliated graphene in the PTP device. The flat probe pushed the hemispherical head of the PTP device at a slow rate of approximately 1 nm/s. Although we could not match the rate at which cracks propagate in the exfoliated graphene, the mechanical properties and structural characteristics of the exfoliated graphene were observed via the tensile tests. The S–S curve was not continuous. The discontinuous curve represents the case where partially discontinuous mechanical behavior occurs during tensile testing (Additional file 9: Movie S3). Each independent graph represents the brittle mechanical properties of graphene layers. The results show that the crack propagation direction along the characteristic edge of the exfoliated graphene may change. This tendency was observed not only in the sample shown in Fig. 4 but also in other exfoliated graphene samples (Additional file 10: Movie S4). Thus, the Young’s modulus of our material exhibits a wide range. Specifically, the Young’s modulus ranged from 89 to 371 GPa, and the maximum stress was 22.3 GPa. These values for exfoliated graphene are smaller than the Young’s modulus of 1 TPa and maximum stress of 130 GPa obtained from AFM indentation experiments and other authors (Lee et al. 2008). This result is consistent with previous reports that fractured graphene exhibits diminished mechanical properties (Suk et al. 2015). We are conducting further research into the S–S curve interpretation of few-layer exfoliated graphene, as well as into the possibility of achieving wrinkle-free exfoliated graphene on the PTP device or enabling the transfer of exfoliated graphene in a monolayer or bilayer.

We also observed the propagation of a series of cracks that originated from the folded area (Fig. 4b-g). We did not confirm existence of pre-cracks in the exfoliated graphene on the PTP device. However, it was natural that the crack propagated from the folded area, which is a known as structural defect (Zhang et al. 2014). Furthermore, spontaneous self-tearing and peeling have been observed in pre-cracked graphene in previous articles (Annett and Cross 2016). The tearing direction of the exfoliated graphene varied by 30 degrees as the angles of the armchair and zigzag edges during tensile testing (Additional file 11: Figure S7). Crack formation in monolayer graphene has been theoretically and experimentally reported to predominantly occur in the direction of the armchair or zigzag edge related with the hexagonal lattice symmetry of graphene and direction of applied tension (Kim et al. 2012). Among these two edge structures, the edge energy of the armchair edge results in longer tear length in strength to crack propagation according to classical fracture theory (Liu et al. 2010). However, in our exfoliated graphene, we can see that torn edges are different from the previous results. An in-depth study of the fracture mechanism in layer structure is required to explain this result.

## Conclusions

A stable, clean, and free-standing exfoliated graphene film was successfully transferred to a PTP MEMS device for in situ TEM tensile testing. The gel material used for transfer was liquefied and penetrated under the exfoliated graphene. Through the results of optical imaging, Raman spectroscopy, and HRTEM imaging, we confirmed that the gel material did not affect the mechanical properties of the exfoliated graphene. Finally, we conducted in situ TEM uniaxial tensile testing, which revealed crack propagation from the folded area in experiments where the S–S curve was obtained simultaneously. Values for the Young’s modulus and maximum stress were also obtained from the S–S curve, and the results were compared with previously reported results. As the crack progressed, we observed that a zigzag or armchair direction corresponding to hexagonal lattice structure appeared at the edge of the exfoliated graphene where the fracture occurred. We envision that our study will lead to the enhanced understanding of the mechanical behavior of 2D materials at an atomic level.

## Additional files

Additional file 1: **Figure S1.** Optical contrast difference method to determine thickness of exfoliated graphene. (a-h) Optical images of 1 L to 8 L exfoliated graphene with a PF film on 300-nm SiO_2_/Si. The scale bars shown in (a–h) are 10 μm. (i) Graph of optical contrast difference in the number of layers in exfoliated graphene. (j) Optical image for three-layer exfoliated graphene. The scale bar is 5 μm. (k) Height profile obtained from the solid line shown in (j). (TIF 793 kb)

Additional file 2: **Figure S2.** The homemade position aligner used to transfer exfoliated graphene onto the region of interest in the PTP device. (TIF 1103 kb)

Additional file 3: **Figure S3.** Optical images for exfoliated graphene on a PTP device (a) before and (b) after the in situ heating Raman experiment. The scale bars are 20 μm. The red crosses indicate the area analyzed by Raman spectroscopy. (c) The Raman spectra before and after the sample was heated at 300 °C. The intensity ratios between the D and G peaks are 0.58 and 0.39, respectively. The remaining peaks shown in the “before” heating result correspond to the peaks from the gel material, which is a proprietary product, so details are omitted. (TIF 487 kb)

Additional file 4: **Movie S1.** In situ heating movie under the optical microscope that the gel material was evaporated when the PTP device was heated to 300 °C. (WMV 3161 kb)

Additional file 5: **Figure S4.** (a) High-angle annular dark field scanning transmission electron microscopy image of the penetrated gel materials under the exfoliated graphene. (b–d) Energy-dispersive X-ray spectroscopy elemental maps of (b) Si, (c) O, and (d) C. The result of element carbon corresponds to penetrated gel materials. The scale bar is 4 nm. (TIF 467 kb)

Additional file 6: **Figure S5.** (a–b) Optical images of the gel materials on or under the exfoliated graphene on a Si substrate. (c–d) Optical images after the gel materials were annealed at 500 °C for 5 min. The scale bar is 50 μm. (TIF 1592 kb)

Additional file 7: **Figure S6.** (a) The result of image processing after the lattice of graphene and the background were removed using software to enhance the gel material. The scale bar is 2 nm. (b) FFT image with the graphene lattice removed by mask filtering. (TIF 655 kb)

Additional file 8: **Movie S2.** In situ TEM tensile test of the exfoliated graphene in the PTP device. (WMV 4208 kb)

Additional file 9: **Movie S3.** Movie for brittle mechanical properties of graphene layers. (WMV 583 kb)

Additional file 10: **Movie S4.** In situ TEM tensile test of other exfoliated graphene sample. (WMV 2388 kb)

Additional file 11: **Figure S7.** TEM image of exfoliated graphene after in situ TEM tensile testing. We matched the orientation of the crack propagation and graphene armchair or zigzag edges through the inset figure SADP. The scale bar is 200 nm. (TIF 1083 kb)

## Abbreviations

2D: Two-dimensional
AFM: Atomic force microscopy
FFT: Fast Fourier transform
FIB: Focused-ion beam
HRTEM: High-resolution transmission electron microscopy
IPA: Isopropyl alcohol
MEMS: Microelectromechanical systems
PET: Polyetylene terephthalate
PTP: Push-to-pull
S-S: Stress-strain
TEM: Transmission electron microscopy
