# Supplementary figures and images for: Dedicated preparation for in situ transmission electron microscope tensile testing of exfoliated graphene
Source: Appl Microsc. 2019 Apr 29;49:3. doi: 10.1007/s42649-019-0005-5 (PMC7818284; doi:10.1007/s42649-019-0005-5)

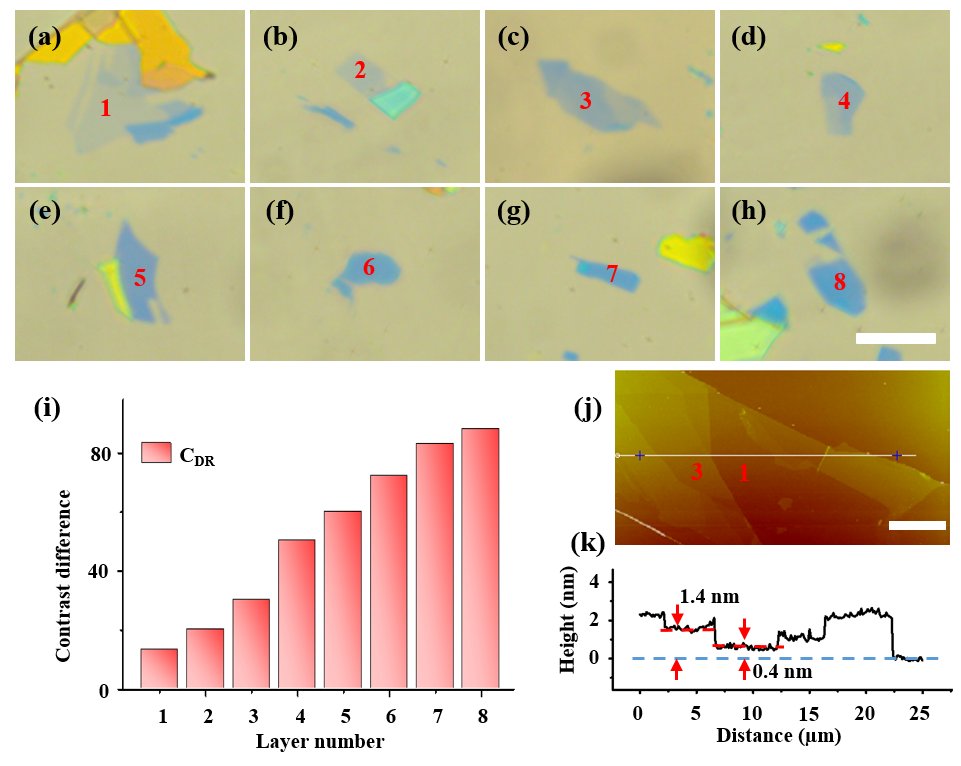

Supplement: Supplementary file 1 — Figure S1. Optical contrast difference method to determine thickness of exfoliated graphene. (a-h) Optical images of 1 L to 8 L exfoliated graphene with a PF film on 300-nm SiO2/Si. The scale bars shown in (a–h) are 10 μm. (i) Graph of optical contrast difference in the number of layers in exfoliated graphene. (j) Optical image for three-layer exfoliated graphene. The scale bar is 5 μm. (k) Height profile obtained from the solid line shown in (j). (TIF 793 kb) [file 42649_2019_5_MOESM1_ESM.tif]

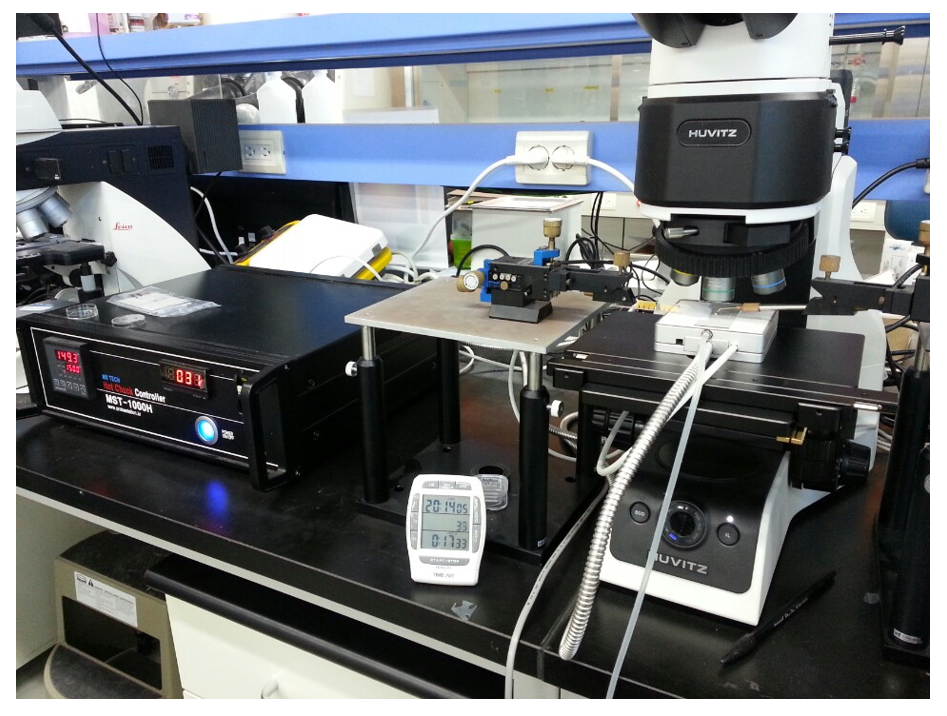

Supplement: Supplementary file 2 — Figure S2. The homemade position aligner used to transfer exfoliated graphene onto the region of interest in the PTP device. (TIF 1103 kb) [file 42649_2019_5_MOESM2_ESM.tif]

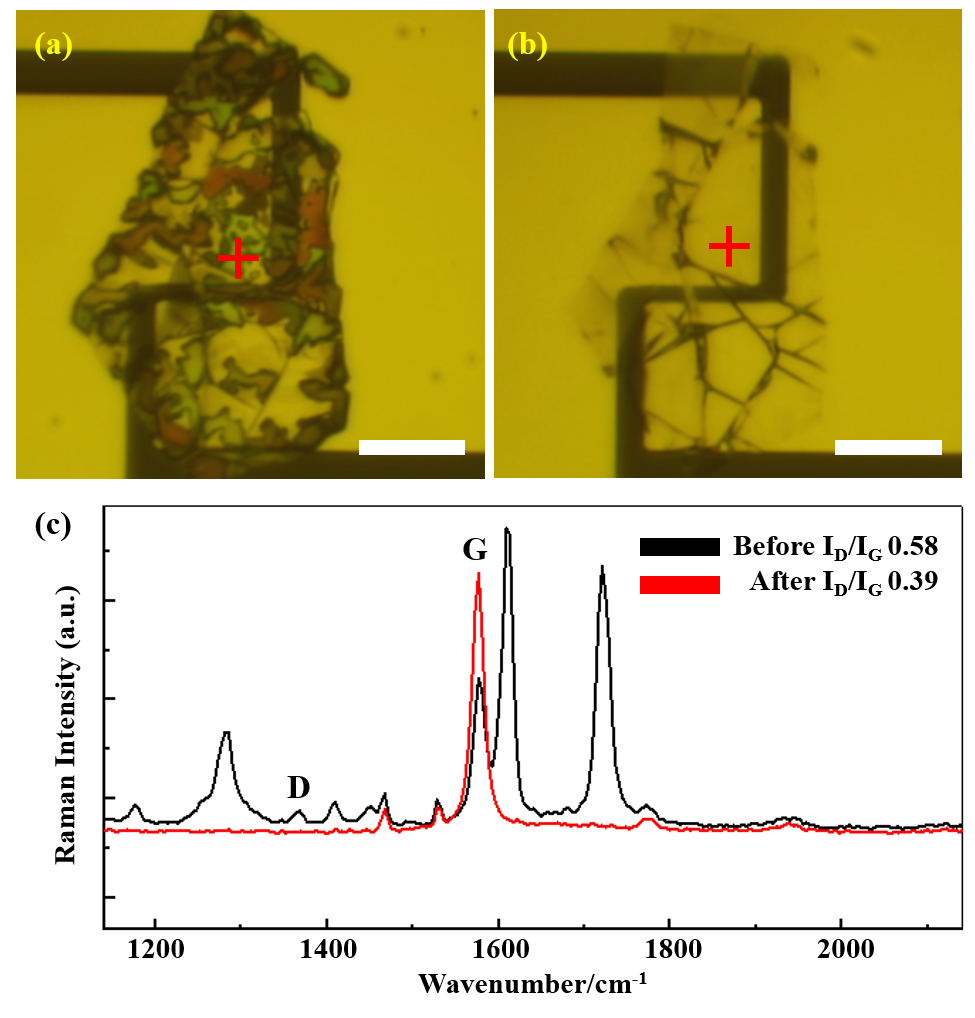

Supplement: Supplementary file 3 — Figure S3. Optical images for exfoliated graphene on a PTP device (a) before and (b) after the in situ heating Raman experiment. The scale bars are 20 μm. The red crosses indicate the area analyzed by Raman spectroscopy. (c) The Raman spectra before and after the sample was heated at 300 °C. The intensity ratios between the D and G peaks are 0.58 and 0.39, respectively. The remaining peaks shown in the “before” heating result correspond to the peaks from the gel material, which is a proprietary product, so details are omitted. (TIF 487 kb) [file 42649_2019_5_MOESM3_ESM.tif]

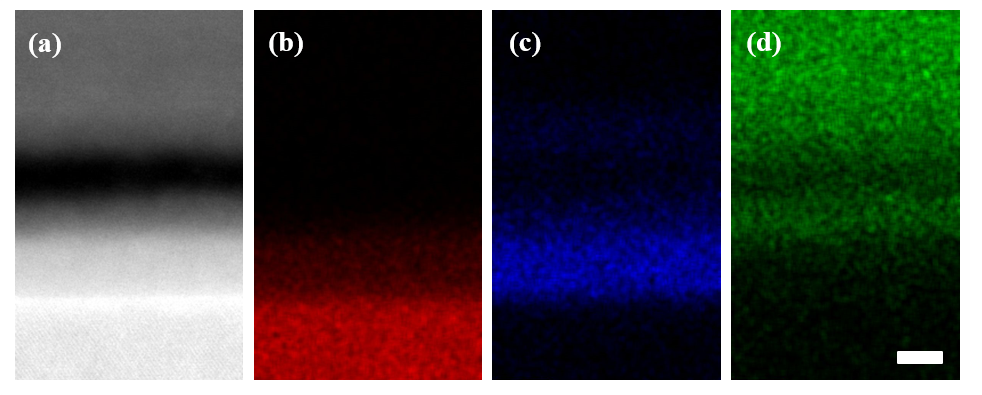

Supplement: Supplementary file 5 — Figure S4. (a) High-angle annular dark field scanning transmission electron microscopy image of the penetrated gel materials under the exfoliated graphene. (b–d) Energy-dispersive X-ray spectroscopy elemental maps of (b) Si, (c) O, and (d) C. The result of element carbon corresponds to penetrated gel materials. The scale bar is 4 nm. (TIF 467 kb) [file 42649_2019_5_MOESM5_ESM.tif]

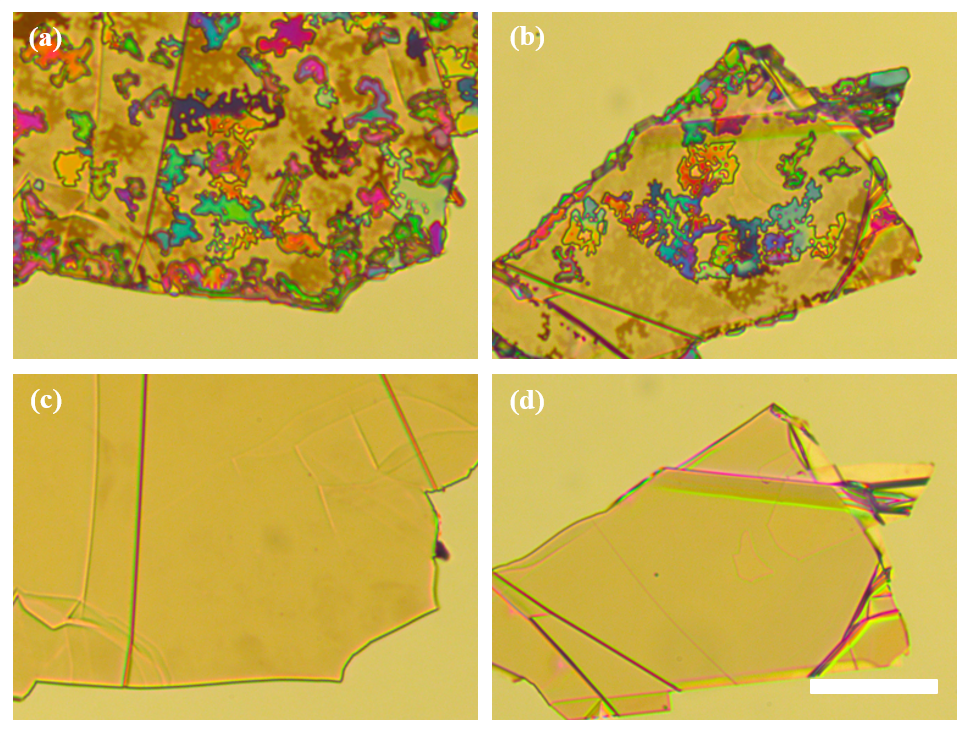

Supplement: Supplementary file 6 — Figure S5. (a–b) Optical images of the gel materials on or under the exfoliated graphene on a Si substrate. (c–d) Optical images after the gel materials were annealed at 500 °C for 5 min. The scale bar is 50 μm. (TIF 1592 kb) [file 42649_2019_5_MOESM6_ESM.tif]

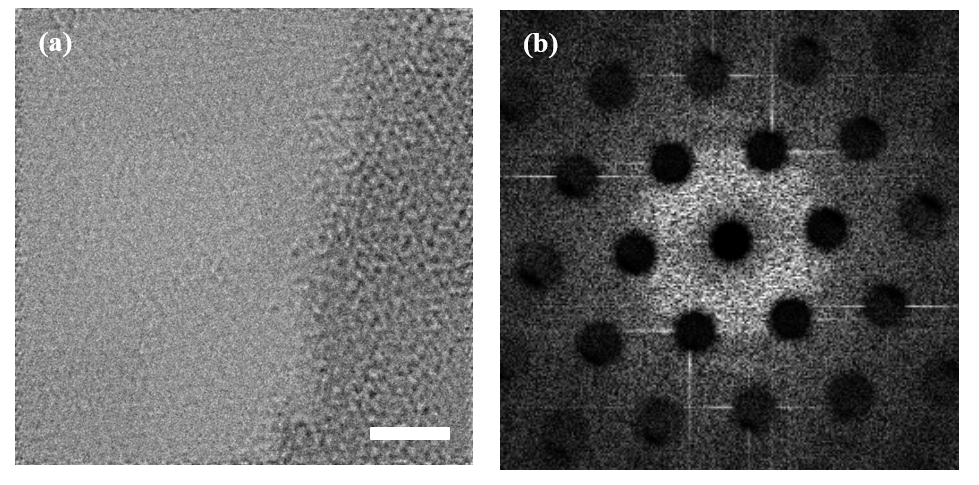

Supplement: Supplementary file 7 — Figure S6. (a) The result of image processing after the lattice of graphene and the background were removed using software to enhance the gel material. The scale bar is 2 nm. (b) FFT image with the graphene lattice removed by mask filtering. (TIF 655 kb) [file 42649_2019_5_MOESM7_ESM.tif]

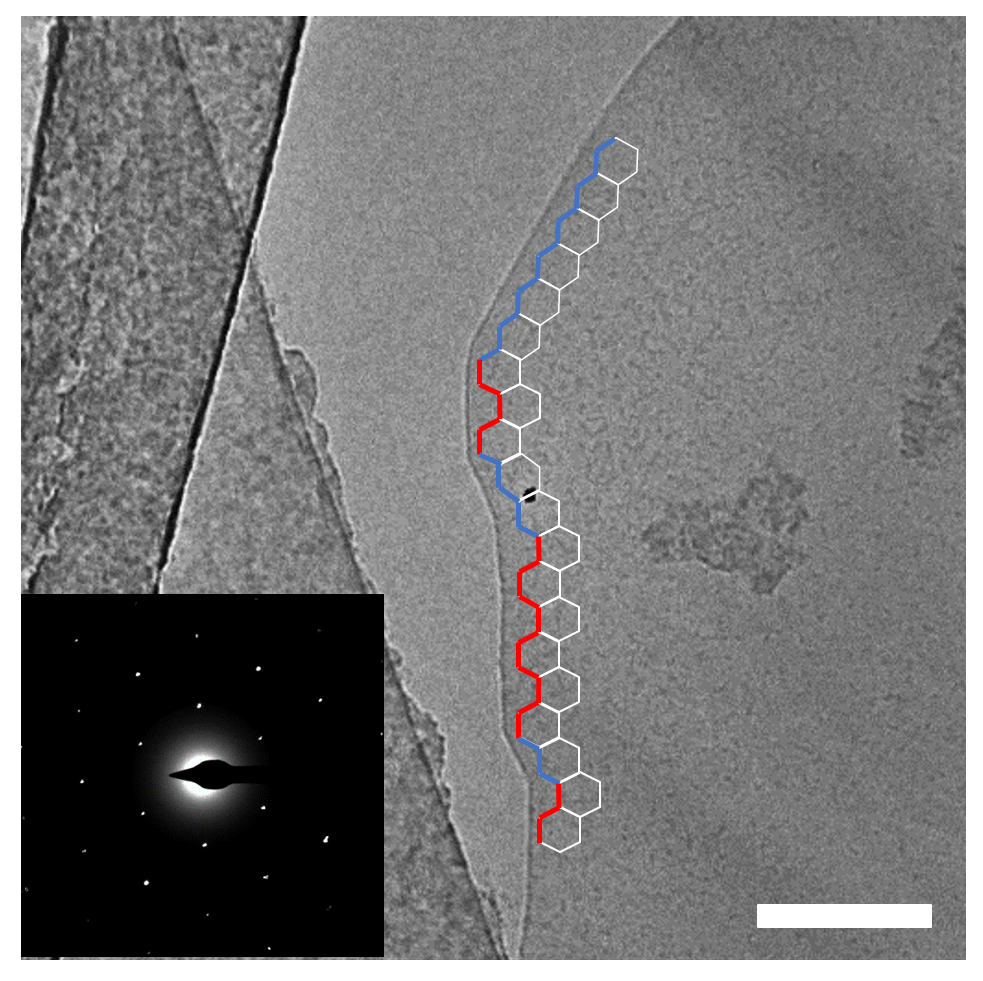

Supplement: Supplementary file 11 — Figure S7. TEM image of exfoliated graphene after in situ TEM tensile testing. We matched the orientation of the crack propagation and graphene armchair or zigzag edges through the inset figure SADP. The scale bar is 200 nm. (TIF 1083 kb) [file 42649_2019_5_MOESM11_ESM.tif]
